# A Rare Case of Pulmonary Alveolar Proteinosis

**DOI:** 10.7759/cureus.90921

**Published:** 2025-08-25

**Authors:** Ayushma Acharya, Adarsha Mahaseth, Sanjiv Poudel, Saurav Paudel, Swarup Sharma Rijal, Roopika Reddy

**Affiliations:** 1 Internal Medicine, Tower Health Medical Group, West Reading, USA; 2 Internal Medicine, Nepalese Army Institute of Health Sciences, Kathmandu, NPL; 3 Internal Medicine, Lakecity Hospital and Critical Care Pvt Ltd., Pokhara, NPL; 4 Internal Medicine, Kist Medical College, Kathmandu, NPL; 5 Medicine, Drexel University College of Medicine, West Reading, USA; 6 Internal Medicine, Reading Hospital Tower Health, West Reading, USA; 7 Pulmonology and Critical Care, Tower Health Medical Group, Wyomissing, USA

**Keywords:** alveolar macrophage dysfunction, autoimmune pulmonary alveolar proteinosis (pap), crazy paving pattern, diffuse parenchymal lung disease, granulocyte-macrophage colony-stimulating factor (gm-csf) autoantibody, pulmonary alveolar proteinosis, recombinant gm-csf therapy, secondary organizing pneumonia, whole lung lavage

## Abstract

Pulmonary alveolar proteinosis (PAP) is a rare surfactant‑clearance disorder most often driven by neutralizing autoantibodies against granulocyte-macrophage colony-stimulating factor (GM-CSF). This leads to accumulation of surfactant in the alveolar spaces, with a symptom spectrum ranging from mild, indolent symptoms to life-threatening respiratory failure. A 50‑year‑old female marathon runner with no smoking history presented with a two-month history of progressively worsening exertional dyspnea and cough. High‑resolution computed tomography (HRCT) showed bilateral peripheral and basal ground‑glass opacities with interlobular septal thickening, producing a "crazy‑paving" pattern. Bronchoalveolar lavage was nondiagnostic; therefore, video‑assisted thoracoscopic surgery (VATS) was performed. Wedge biopsy demonstrated alveolar periodic acid‑Schiff-positive material, and serum GM-CSF autoantibody concentration was 49.9 µg/mL (reference < 3 µg/mL), confirming autoimmune PAP. Whole lung lavage (WLL) yielded transient clinical and radiographic improvement, but relapse occurred within three months, with diffusing capacity of the lungs for carbon monoxide (DLCO) falling to 36% predicted. Daily subcutaneous followed by inhaled recombinant GM-CSF (sargramostim 5 µg/kg) provided lasting symptom relief, reduced ground-glass lung infiltrates, and improved DLCO to 65% of predicted. PAP should be suspected in progressive respiratory failure with typical imaging findings, particularly when no secondary cause is identified. For patients who do not respond to WLL or recombinant GM-CSF, further studies are needed to define the best treatment approach.

## Introduction

Pulmonary alveolar proteinosis (PAP) is a rare lung disorder characterized by impaired alveolar macrophage function, resulting in the accumulation of excess surfactant within the alveolar spaces [1]. First described by Rosen and colleagues in 1958 [2], PAP remains uncommon, with an estimated incidence of approximately 0.2 cases per million individuals per year [3]. Based on underlying pathophysiology, PAP is classified into three types: congenital, secondary, and autoimmune (also referred to as acquired or idiopathic) PAP [4]. The autoimmune form, caused by autoantibodies against granulocyte-macrophage colony-stimulating factor (GM-CSF), accounts for approximately 90% of all cases [5]. All forms lead to impaired surfactant clearance and progressive impairment in gas exchange [6]. In autoimmune PAP, impaired GM-CSF signaling plays a central role. GM-CSF normally drives maturation of alveolar macrophages and surfactant catabolism, while its neutralization by autoantibodies leads to macrophage dysfunction and surfactant accumulation. Clinical manifestations arise from widespread surfactant overload within alveoli and range from mild, indolent symptoms to life-threatening respiratory failure [7].

Many patients remain asymptomatic at the time of diagnosis, and a substantial proportion of cases are detected incidentally [8]. When symptoms are present, they are often nonspecific, with dyspnea and cough (either productive or nonproductive) being the most frequent complaints [8,9]. Less frequently, patients may experience chest pain, weight loss, fatigue, fever, and hemoptysis. The presence of hemoptysis should prompt evaluation for superimposed infection or other complicating factors [9]. High-resolution computed tomography (HRCT) of the chest typically reveals a characteristic “crazy-paving” pattern, defined by ground-glass opacities overlaid with interlobular septal thickening. Although this radiographic finding is suggestive of PAP, it is not pathognomonic and can be seen in a variety of other pulmonary conditions [10,11]. Definitive diagnosis of PAP requires identification of periodic acid-Schiff (PAS)-positive, lipoproteinaceous material within the alveolar spaces on lung biopsy or bronchoalveolar lavage specimens, in conjunction with elevated serum level of anti-GM-CSF autoantibodies [6,12].

We herein report a case of a 50-year-old female, a nonsmoker and competitive marathon runner, who developed progressive dyspnea and was ultimately diagnosed with autoimmune PAP.

## Case presentation

A 50-year-old nonsmoking woman and competitive marathon runner first noted progressive shortness of breath and a persistent, nonproductive cough over a two-month period. She reported occasional whitish sputum but denied fever, chills, hemoptysis, chest pain, weight loss, or night sweats, which suggested a chronic noninfectious process rather than acute infection or malignancy. Her past medical history was notable for mild iron-deficiency anemia, and she was not taking any regular medications. Her past medical history was notable only for mild iron-deficiency anemia, and she had no family history of pulmonary disease. She reported significant occupational exposure in a mold-damaged school building, raising the possibility of environmental contribution. On physical examination, she appeared comfortable at rest, and her vital signs were as follows: blood pressure at 118/72 mmHg, heart rate of 78 beats per minute, respiratory rate of 16 breaths per minute, and oxygen saturation of 97% on room air. Pulmonary auscultation revealed fine bibasilar crackles, which pointed to an underlying interstitial lung process. The remainder of the examination was unremarkable.

**Figure 1:**
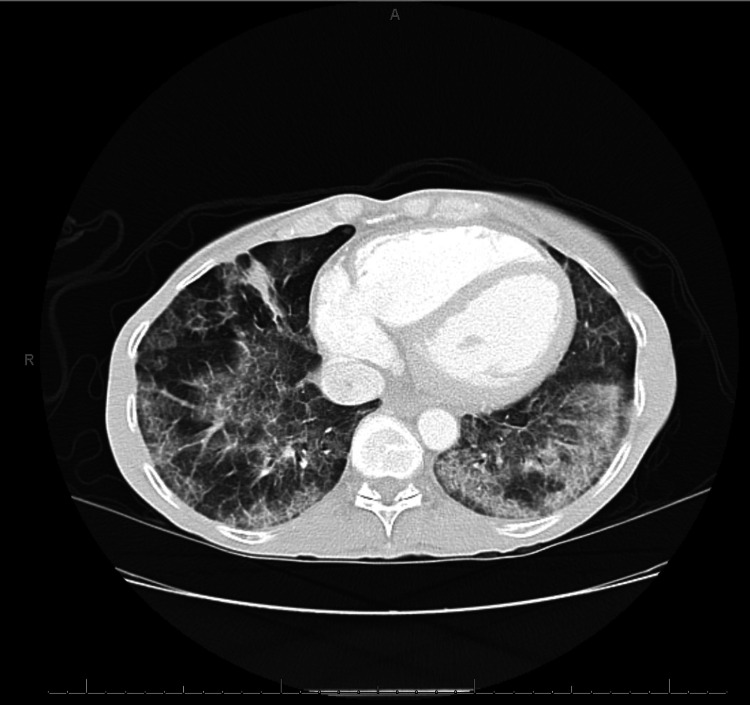
High-resolution axial CT of the chest at the basal level, demonstrating bilateral, predominantly peripheral ground-glass opacities with superimposed interlobular septal thickening (the classic “crazy-paving” pattern) consistent with pulmonary alveolar proteinosis. The diffuse ground-glass opacities and interlobular septal thickening seen on high-resolution computed tomography ("crazy-paving") correlate with her severe exertional dyspnea and reduced diffusing capacity of the lungs for carbon monoxide, reflecting impaired alveolar gas exchange.

Initial chest radiography demonstrated bilateral reticulonodular infiltrates, most prominent in the lower lung zones. HRCT of the chest confirmed diffuse, peripheral, and basally predominant ground-glass opacities coupled with interlobular septal thickening, findings characteristic of the “crazy-paving” pattern, without evidence of honeycombing or traction bronchiectasis (Figure 1). Pulmonary function testing revealed a normal forced expiratory volume in one second (FEV₁)/forced vital capacity (FVC) ratio, mild restrictive pattern (total lung capacity: 85% predicted), and a markedly reduced diffusing capacity of the lungs for carbon monoxide (DLCO: 12.4 mL/min/mmHg, 54% of predicted), with only partial correction for alveolar volume. The results of the comprehensive metabolic panel are presented in Table 1.

**Table 1:** Comprehensive metabolic panel results. Reference ranges reflect our laboratory standards. All measured values are within or near normal limits. Mildly low albumin (3.4 g/dL) is not clinically significant in the context of this case. Overall, metabolic parameters do not suggest systemic organ dysfunction contributing to the pulmonary findings.

Parameter	Patient value	Reference range
Glucose (mg dL-¹)	77	70 – 99
Blood urea nitrogen (mg dL-¹)	8	7 – 20
Creatinine (mg dL-¹)	0.70	0.6 – 1.3
Estimated glomerular filtration rate (mL min-¹ 1.73 m²)	88.6	≥60
Sodium (mmol L-¹)	142	135 – 145
Potassium (mmol L-¹)	3.9	3.5 – 5.0
Chloride (mmol L-¹)	111	96 – 106
Bicarbonate (mmol L-¹)	29	22 – 30
Calcium (mg dL-¹)	8.5	8.6 – 10.2
Anion gap (mmol L-¹)	2	8 – 16
Osmolality (mOsm kg-¹)	291	275 – 295
Albumin (g dL-¹)	3.4	3.5 – 5.0
Total protein (g dL-¹)	6.3	6.4 – 8.3
Total bilirubin (mg dL-¹)	0.4	0.1 – 1.2
Alkaline phosphatase (U L-¹)	83	44 – 147
Alanine transaminase (U L-¹)	46	7 – 56
Aspartate transaminase (U L-¹)	36	5 – 40

Comprehensive rheumatologic panel was negative (Table 2). Bronchoscopy with bronchoalveolar lavage (BAL) and transbronchial biopsy was performed to further investigate the underlying etiology. The lavage fluid appeared opaque and tan-pink to gray-white. The BAL differential showed a low neutrophil proportion (21%, reference range: 40-75%), with other values within normal limits (Table 3).

**Table 2:** Rheumatologic antibody panel. NEG = negative; IU mL⁻¹ = international units per milliliter. Reference ranges reflect our laboratory standards. All autoantibodies are negative, ruling out underlying connective tissue disease or autoimmune etiology other than autoimmune pulmonary alveolar proteinosis.

Parameter	Patient value	Reference range
Smith (Sm) antibody	<1.0 NEG	<1.0
Sm/ribonucleoprotein antibody	<1.0 NEG	<1.0
Sjögren’s antibody (SSA/Ro)	<1.0 NEG	<1.0
Sjögren’s antibody (SSB/La)	<1.0 NEG	<1.0
Centromere B antibody	<1.0 NEG	<1.0
Antineutrophil cytoplasmic antibody (ANCA)	Negative	Negative
Rheumatoid factor (IU/mL)	<14	<20
Cyclic citrullinated peptide antibody (units)	<16	<20

**Table 3:** Bronchoalveolar lavage (BAL) differential cell count. Reference ranges reflect our laboratory standards. CD4/CD8: helper-to-suppressor T-cell ratio. Neutrophil proportion is below normal, suggesting impaired alveolar immune function. Monocytes are markedly elevated, consistent with alveolar macrophage accumulation typical of pulmonary alveolar proteinosis (PAP). Other cell types are within reference ranges, and red blood cells indicate only mild non-hemorrhagic contamination.

Parameter	Patient value	Reference range	Notes
White blood cell count	181 cells/mm³	<200 cells/mm³	Within normal limits
Neutrophils	21%	40–75%	Below reference range
Lymphocytes	29%	20–45%	Within normal range
Monocytes	39%	2–10%	Markedly elevated
Eosinophils	Not significant	<2%	Not elevated
Red blood cells	<2000 cells/mm³	<3000 cells/mm³	Mild RBC presence; not hemorrhagic
CD4:CD8 ratio (BAL)	1.4	1.0–2.0	Normal

Microbiologic studies, including bacterial, mycobacterial (acid-fast bacilli smear and culture), and fungal cultures, were all negative. Cytology analysis showed no evidence of malignancy. Transbronchial biopsy specimens demonstrated preserved alveolated lung parenchyma without significant inflammation or fibrosis. A four-week tapering course of systemic corticosteroids, initiated at prednisone 40 mg daily, yielded no clinical or radiographic improvement.

Given ongoing clinical suspicion for a diffuse parenchymal lung disease, a video-assisted thoracoscopic (VATS) wedge biopsy was performed. Macroscopically, the lung surface appeared indurated and granular. Histologic sections revealed alveolar spaces filled with homogeneous, granular eosinophilic material that stained strongly with PAS and was resistant to diastase digestion. Focal areas of organizing pneumonia were also observed in adjacent tissue. These findings established the diagnosis of PAP. Subsequent serum testing demonstrated markedly elevated anti-GM-CSF autoantibody levels at 49.9 µg/mL (reference < 3 µg/mL), confirming a diagnosis of autoimmune PAP.

The patient underwent sequential whole-lung lavages, first of the right lung, followed by the left, under general anesthesia with double-lumen endotracheal intubation. She experienced immediate symptomatic relief, and follow-up chest HRCT revealed a marked reduction in ground-glass opacities. However, three months post lavage, she developed recurrent exertional dyspnea. Repeat chest HRCT demonstrated reappearance of bilateral ground-glass infiltrates, and pulmonary function testing revealed an isolated decline in DLCO to 36% of predicted (Table 4).

**Table 4:** Pulmonary function test (PFT) metrics. FVC = forced vital capacity; FEV₁ = forced expiratory volume in one second; VC = vital capacity; RV = residual volume; TLC = total lung capacity; DLCO = diffusing capacity of the lung for carbon monoxide. % predicted values are calculated from age-, sex-, height-, and ethnicity-specific reference equations. * 70% denotes the conventional lower limit of normal for the FEV₁/FVC ratio, not a percent predicted value. FVC and VC show mild restriction. DLCO is severely reduced (36% predicted), consistent with impaired gas exchange and correlating with the patient’s recurrent exertional dyspnea. FEV₁/FVC ratio remains normal, indicating preserved airway patency.

Parameter	Absolute value	% Predicted/criterion	Interpretation
FVC (L)	2.80	75%	Mild restriction
FEV₁ (L)	2.35	80%	Borderline normal
FEV₁/FVC (%)	84	≥70% expected*	Normal ratio
VC (L)	2.58	76%	Mild restriction
RV (L)	1.56	85%	Normal
TLC (L)	4.04	77%	Mild restriction
DLCO	36% predicted	-	Severe diffusion defect

Given disease recurrence despite lavage, subcutaneous sargramostim (recombinant GM-CSF) was initiated at a dose of 250 µg daily (5 µg/kg). After eight weeks of subcutaneous therapy, she transitioned to inhaled GM-CSF (250 µg twice daily) for improved convenience. Treatment was well tolerated, with only mild, transient injection-site erythema. Serial assessments at three and six months demonstrated progressive improvement in gas exchange, with DLCO rising to 65% of predicted. Exercise tolerance also improved, with the patient able to jog 3 km while maintaining a nadir oxygen saturation of 92%. Imaging showed partial resolution of opacities without evidence of new fibrotic changes.

During this period, the patient remained under close multidisciplinary follow-up, with regular clinical evaluations, pulmonary function tests, and imaging. She reports significant improvement in quality of life, has resumed moderate exercise, and experiences minimal respiratory limitation.

## Discussion

PAP is an ultra-rare disorder characterized by intra-alveolar accumulation of surfactant lipoproteins [1]. The demographic profile of our patient was notably atypical. PAP most commonly affects otherwise healthy adults between the ages of 30 and 50 years, with a male predominance and a strong association with smoking history [13]. In contrast, our patient was a 50-year-old female, a nonsmoker and competitive endurance athlete. This case highlights that autoimmune PAP can present even in low-risk, fit individuals. Current guidelines advise that when chest imaging in an otherwise healthy individual aged 30-50 years demonstrates features suggestive of PAP, such as a crazy-paving pattern, clinicians should include PAP in the differential diagnosis [14].

Imaging and initial bronchoscopy reflected classic features of PAP. Chest HRCT demonstrated diffuse ground-glass opacities with superimposed septal thickening, creating the characteristic “crazy-paving” pattern. These changes result from alveolar surfactant accumulation caused by impaired macrophage-mediated clearance [6]. Although this pattern is not exclusive to PAP, it is highly suggestive of PAP in the appropriate clinical context. BAL yielded opaque, milky (tan-pink) fluid that was PAS-positive. This appearance indicates the presence of surfactant-rich material within the alveoli and demonstrates impaired surfactant clearance by alveolar macrophages [6]. However, transbronchial biopsies were non-diagnostic - a known limitation in PAP [15]. Current guidance recommends proceeding to surgical lung biopsy when BAL findings are inconclusive, as was necessary in this case [16]. In a retrospective case series of four patients treated for PAP at the State University of Londrina School of Medicine, the final diagnosis was established via open-lung biopsy after nondiagnostic BAL [17]. Similarly, a multicentric retrospective study of primary PAP in India found that when transbronchial lung biopsy was inconclusive, diagnosis was confirmed via transbronchial lung cryobiopsy or surgical lung biopsy [18].

A definitive diagnosis was established through histopathology and serology. VATS lung biopsy demonstrated alveoli filled with eosinophilic, PAS-positive proteinaceous material, hallmark features of PAP [4,19]. Interestingly, focal areas of organizing pneumonia were also identified in adjacent lung parenchyma. Although not a classic PAP finding, organizing pneumonia has been reported in selected cases, particularly in association with hematologic disorders [20], suggesting that overlapping inflammatory processes are involved. Crucially, serologic testing revealed markedly elevated anti-GM-CSF autoantibodies (49.9 µg/mL, normal <3 µg/mL), confirming the autoimmune subtype. Nearly all patients with autoimmune PAP demonstrated high-titer anti-GM-CSF antibodies [21], and their presence is considered diagnostic, with reported sensitivity and specificity of 100% and 98%, respectively [22]. The patient’s markedly elevated antibody level, well above the approximate 5 µg/mL threshold observed in healthy individuals [23], confirmed the diagnosis.

Following diagnosis, standard therapy for symptomatic PAP was initiated. Whole lung lavage (WLL) under general anesthesia remains the cornerstone of treatment [24]. The patient underwent sequential bilateral WLL, resulting in immediate symptom relief. WLL is well established to improve oxygenation and reduce dyspnea. In one large case series, over 70% of patients remained recurrence-free for more than seven years following WLL [25]. However, the patient experienced a relapse within three months, highlighting a known limitation of lavage monotherapy in autoimmune PAP; continued autoantibody production typically leads to surfactant re-accumulation over time. Repeat WLLs are often needed unless the underlying GM-CSF deficiency is addressed [13].

In response to the early relapse, GM-CSF replacement therapy was initiated. Both subcutaneous [26] and inhaled [27] GM-CSF have demonstrated clinical benefit in autoimmune PAP. Initial trials using subcutaneous GM-CSF reported response rates of approximately 40-50% [23], while more recent studies of inhaled therapy have improved efficacy. A pooled analysis reported an 80% overall response rate, with superior oxygenation improvements and lower relapse rates observed with inhaled versus subcutaneous administration [28]. The patient initially received eight weeks of subcutaneous sargramostim before transitioning to inhaled dosing (250 µg twice daily), which was well tolerated. Over the subsequent three to six months, she experienced significant clinical and physiological improvements, including increased DLCO, enhanced exercise tolerance, and radiographic resolution of ground glass opacities. These outcomes are consistent with previously published data: in one case series, patients receiving inhaled GM-CSF exhibited durable responses without further need for WLL [29]. This case supports these findings and suggests that a sequential delivery strategy, beginning with subcutaneous administration, followed by inhaled therapy, may offer both convenience and therapeutic benefit.

Comprehensive longitudinal monitoring, including serial pulmonary function tests, repeat HRCTs, and clinical exams, was performed to track treatment response and detect recurrence. This approach aligns with current guidelines, which recommend structured follow-up every six months to assess symptoms, gas exchange, and imaging findings [30]. In this patient, follow-up demonstrated progressive improvement in DLCO and symptom burden, without evidence of fibrotic progression on imaging, with other reports of GM-CSF therapy, and no fibrotic progression on imaging, which further supports the long-term benefit of inhaled GM-CSF therapy in autoimmune PAP.

Distinctive features of this case

Athletic, Nonsmoking Patient

In contrast to the typical PAP demographic profile, such as middle-aged, male, and often with a history of smoking, our patient was a nonsmoker and a competitive marathon runner. This suggests that autoimmune PAP can occur in individuals with no identifiable risk factors and high baseline cardiopulmonary fitness.

“Crazy-Paving” Pattern With Non-diagnostic Bronchoscopy

The patient's HRCT showed the classic crazy-paving pattern, and BAL fluid was characteristically milky, raising early suspicion for PAP. However, transbronchial biopsies were nondiagnostic, highlighting the known limitations of BAL and the importance of proceeding to surgical biopsy when initial investigations are inconclusive.

High-Titer Anti-GM-CSF Antibody

Serological testing showed a markedly elevated level of anti-GM-CSF autoantibodies (49.9 µg/mL). Levels >5 µg/mL are typically seen only in autoimmune PAP [23], and such high titers are diagnostic of the autoimmune form.

Incidental Organizing Pneumonia

Histological examination revealed focal organizing pneumonia adjacent to the areas of alveolar proteinosis. While organizing pneumonia is not a typical feature of PAP, rare cases have been described, particularly in association with underlying hematological or inflammatory disorders. This finding suggests a possible overlapping or secondary inflammatory process.

Rapid Recurrence Post-lavage

Despite an adequate bilateral WLL (the standard therapy), the patient experienced relapse. While many patients experience long-term remission after lavage, this case highlights that relapse can occur soon after, reflecting the persistent autoimmune drive.

Sequential GM-CSF Therapy

We employed subcutaneous then inhaled GM-CSF (sargramostim) with close monitoring. The patient experienced measurable gains in gas transfer and exercise capacity, aligning with literature showing high response rates (≈80%) to inhaled GM-CSF and improved oxygenation indices.

Long-Term Multidisciplinary Follow-Up

Over six months of follow-up, the patient had multidisciplinary care with regular PFTs, imaging, and clinical review. This approach, recommended in recent guidelines, allowed us to tailor therapy and avoid complications. The patient’s quality of life significantly improved, and she returned to moderate exercise with minimal limitations.

## Conclusions

This case highlights the broad clinical spectrum of autoimmune PAP. Even highly fit, asymptomatic individuals may harbor significant disease, and the presence of classic imaging clues, such as a “crazy-paving” pattern, should prompt a thorough and timely diagnostic workup. While serum anti-GM-CSF autoantibody testing now permits less invasive confirmation of autoimmune PAP, as demonstrated in our case, tissue biopsy may still be necessary when BAL findings are equivocal. This case also reinforces current management principles: WLL remains the first-line treatment for symptomatic relief, but combination therapy with GM-CSF, particularly in its inhaled form, and vigilant clinical follow-up often provides the most favorable long-term outcome.
